# Aging and death-associated changes in serum albumin variability over the course of chronic hemodialysis treatment

**DOI:** 10.1371/journal.pone.0185216

**Published:** 2017-09-27

**Authors:** Yuichi Nakazato, Riichi Kurane, Satoru Hirose, Akihisa Watanabe, Hiromi Shimoyama

**Affiliations:** 1 Division of Nephrology, Yuai Nisshin Clinic, Hakuyukai Medical Corporation, Saitama-City, Saitama, Japan; 2 Division of Nephrology, Yuai Clinic, Hakuyukai Medical Corporation, Saitama-City, Saitama, Japan; 3 Division of Nephrology, Yuai Mihashi Clinic, Hakuyukai Medical Corporation, Saitama-City, Saitama, Japan; 4 Division of Nephrology, Yuai Nakagawa Clinic, Hakuyukai Medical Corporation, Saitama-City, Saitama, Japan; Universidade Estadual Paulista Julio de Mesquita Filho, BRAZIL

## Abstract

**Background:**

Several epidemiological studies have demonstrated associations between variability in a number of biological parameters and adverse outcomes. As the variability may reflect impaired homeostatic regulation, we assessed albumin variability over time in chronic hemodialysis (HD) patients.

**Methods:**

Data from 1346 subjects who received chronic HD treatment from May 2001 to February 2015 were analyzed according to three phases of HD treatment: post-HD initiation, during maintenance HD treatment, and before death. The serum albumin values were grouped according to the time interval from HD initiation or death, and the yearly trends for both the albumin levels and the intra-individual albumin variability (quantified by the residual coefficient of variation: Alb-rCV) were examined. The HD initiation and death-associated changes were also analyzed using generalized additive mixed models. Furthermore, the long-term trend throughout the maintenance treatment period was evaluated separately using linear regression models.

**Results:**

Albumin levels and variability showed distinctive changes during each of the 3 periods. After HD initiation, albumin variability decreased and reached a nadir within a year. During the subsequent maintenance treatment period (interquartile range = 5.2–11.0 years), the log Alb-rCV showed a significant upward trend (mean slope: 0.011 ± 0.035 /year), and its overall mean was -1.49 ± 0.08 (equivalent to an Alb-rCV of 3.22%). During the 1–2 years before death, this upward trend clearly accelerated, and the mean log Alb-rCV in the last year of life was -1.36 ± 0.17. The albumin levels and variability were negatively correlated with each other and exhibited exactly opposite movements throughout the course of chronic HD treatment. Different from the albumin levels, albumin variability was not dependent on chronological age but was independently associated with an individual’s aging and death process.

**Conclusion:**

The observed upward trend in albumin variability seems to be consistent with a presumed aging-related decline in homeostatic capacity.

## Introduction

Several observational studies on blood pressure, blood glucose, and blood hemoglobin have shown associations between a high variability in these parameters and an adverse outcome [1–6]. We recently examined the variability of these and other parameters in routine blood examinations of hemodialysis (HD) patients and found that such associations are not limited to only a few parameters. Variability in urea nitrogen, sodium, hemoglobin, creatinine, albumin, potassium, phosphate and others were often, but differently, associated with a poor survival outcome, impaired mobility, and other markers of a poor prognosis, including hypoalbuminemia and hyponatremia [7]. Some studies have shown an elevated intra-individual variability in these laboratory parameters in patients with certain chronic diseases [8–11]. In addition, for patients with chronic kidney disease (CKD), the variability of hemoglobin and blood pressure has been reported to increase according to the CKD stage [12–14].

These observations in general indicate that variability is related to an unhealthy status. In this regard, it should be noted that frailty, an aging-related and unhealthy condition, is often described as “a syndrome associated with a limited capacity to maintain homeostasis” [15,16]. Similar to the extent of variability, the prevalence of frailty is known to be high in populations with chronic diseases, particularly advanced CKD [17,18]. Considering these facts, a diminished homeostatic control is likely reflected by an elevated variability of some biological parameters. If our assumption is correct, the magnitude of the variability should increase with aging or a deterioration in health conditions [19]. In this study, we examined the longitudinal changes in serum albumin variability during the course of maintenance HD treatment.

## Methods

### Study cohort

**Fig 1:**
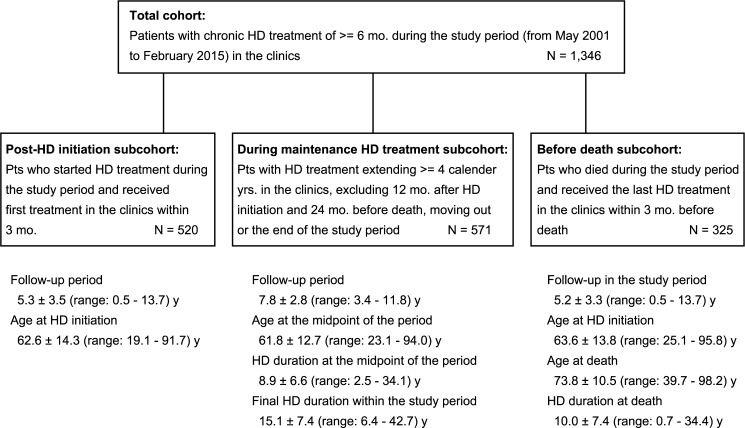
Patient selection.

A total of 1346 patients (31.1% female, 42.1% diabetic) received chronic HD treatment for more than 6 months between May 2001 and January 2015 (study period) at 4 outpatient HD facilities in Saitama-City, Japan. Most of them underwent long-term HD treatment, and the median of the final HD duration within the study period was 7.6 years (interquartile range = 4.0–13.7 years). We retrospectively analyzed the serum albumin dynamics in appropriately selected cohort subsets during 3 phases of maintenance HD treatment: i) the post-HD initiation period, ii) during maintenance HD treatment, and iii) before death (Fig 1).

#### Post-HD initiation subcohort

Of our cohort, 520 patients had started regular HD treatment at one of the study facilities within 3 months of the first dialysis treatment session occurring during the study period. The mean (± SD) patient age at HD initiation was 62.6 ± 14.3 years; 29.6% of the patients were female, and 48.8% were diabetic.

#### Before death subcohort

A total of 325 patients died while being treated at the study facilities during the study period or within 3 months of their last HD treatment at the study facilities during the study period. The mean (± SD) patient ages at HD initiation and at death were 63.6 ± 13.8 and 73.7 ± 10.5 years, respectively. The mean HD duration at the time of death was 10.1 ± 7.4 years.

#### During maintenance HD treatment subcohort

Based on our analyses for the post-HD initiation and before death subcohorts, we defined the maintenance period of HD treatment for each patient as that beginning 1 year after HD initiation and ending 2 years before the censored time (transfer, death, or the end of the study period). For each patient, only calendar years that contained 6 or more serum albumin determinations performed during more than 6 months of HD treatment during the year were regarded as containing sufficient data, and the data for these calendar years were included in the study. To determine the long-term trend in albumin levels and variability in a reliable manner, the subjects were limited to 571 patients who had data for more than 4 eligible calendar years during the maintenance period of HD treatment. Consequently, most of the subjects were long-term survivors, and the mean length of the maintenance period was 7.8 ± 2.8 years. The HD duration at the middle point of the maintenance period was 8.9 ± 6.6 years (Fig 1).

This retrospective observational study was approved by the institutional ethics committee of Hakuyukai Medical Corporation (approval number: 27–001) and was conducted in accordance with the principles of the Declaration of Helsinki. Informed consent was provided from all the patients who underwent HD treatment in the facilities during 2015.

### Data collection and calculations

**Fig 2:**
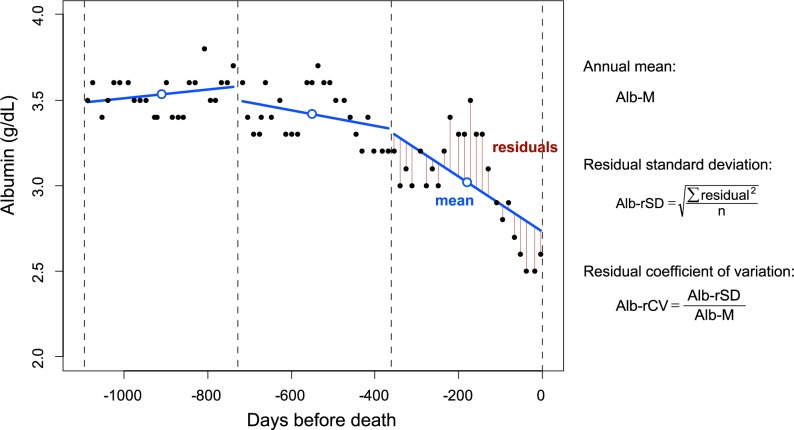
Evaluation of intra-individual albumin variability. The serum albumin values in a representative case were plotted over the 3 years before death. The values measured during one year were fitted to a linear regression model, and the residual SD and the residual CV were calculated using the indicated formulas.

All the subjects underwent a regular blood examination twice a month. During these regular examinations, the serum albumin level was measured 6–8 times per year until the end of 2006 and 24 times per year thereafter. The blood samples from the study facilities were analyzed at a single external laboratory, and the serum albumin level was measured using the bromcresol green method. The laboratory and demographic data were retrieved from electronic databases. As serum albumin levels and other blood parameters exhibit seasonal changes [20], the albumin levels and their variability were estimated, in principal, on a yearly basis for each subject. A period of 1 year was determined by either the calendar time or the interval from the date of HD initiation or death. If the number of albumin determinations per year was less than 6 for an individual patient, the data for that year was excluded from the yearly analysis. The albumin level was represented by its yearly mean value and was abbreviated as Alb-M. Albumin variability was defined by the coefficient of variation (CV = standard deviation/mean) or the derived coefficient, residual CV (= residual standard deviation/mean) [5,21] (Fig 2). As the serum albumin level showed a significantly increasing (or decreasing) trend during the periods following HD initiation or prior to death, the CV could overestimate the variability during these periods. Therefore, we eventually used the residual CV as an index of variability in this longitudinal study. The residual CV of serum albumin (Alb-rCV) was log10 transformed to normalize its distribution for the statistical analysis.

### Statistical analysis

All the analyses were performed in R 3.1.2. (R Core Team, 2014) using the gplots, mgcv, and gamm4 packages. The changes in the albumin levels (Alb-M) and the variability (log Alb-rCV) during the post-HD initiation and before death periods were statistically determined on a yearly basis for patients who had received continuous HD treatment for more than 3 years during either period. Differences between years were detected at a level of significance of 0.05 by pairwise comparisons using paired t-test with Holm’s adjustment.

In addition to the yearly analysis, shorter-term changes in albumin levels were assessed using generalized additive mixed models [22]. We fitted all the measured albumin values into a random intercept model including the time interval as a fixed effect and the patient identifier as a random effect. In a similar fashion, temporal changes in albumin variability were assessed by fitting moving CV values (instead of log residual CV values) into the same model. The moving CV (= moving standard deviation / moving average) was calculated for a moving window containing 3 consecutive albumin values and was treated as a variability index on the middle day. While a moving CV mirrors temporal changes better than a yearly calculated Alb-rCV, it can lead to an overestimation of variability if the source data has a continuous trend and particularly if it is sampled at a long interval. Therefore, only moving CV values for the year 2007 or thereafter were used in the model. In this setting, a constant decrease in albumin values, from 3.50 to 3.24 mg/dL in one year, was estimated to increase the CV by 0.26%.

An individual general trend for Alb-M (or log Alb-rCV) during the maintenance period of HD treatment was estimated using a linear regression model fitted for the yearly calculated Alb-M (or log Alb-rCV) values. As the ends of the maintenance period usually did not coincide with those of the calendar years, the first or last calendar year of the maintenance period contained fewer albumin data points. Considering the lower reliability of the Alb-M and log Alb-rCV values in the boundary years, both values were weighted with the duration of HD treatment within the year; if the duration was shorter than 6 months, the values were discarded. The slopes of the obtained regression lines were then applied to one sample t-test to determine whether their mean value was significantly different from zero.

## Results

### Serum albumin dynamics before death

As previously reported, the albumin levels of individual patients often showed a downward trend before death. At the same time, we noticed an increase in fluctuations toward death in several subjects. A representative case is shown in Fig 2.

**Fig 3:**
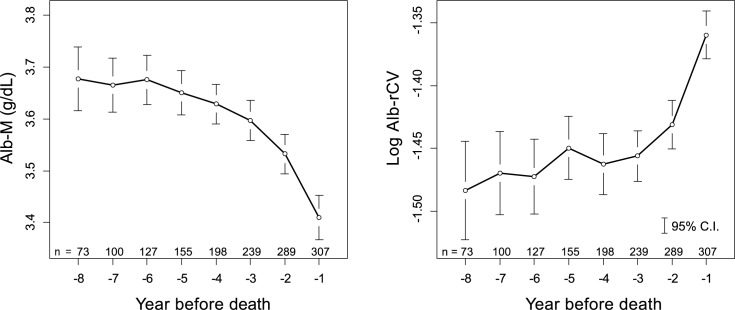
Yearly trend in albumin levels and variability prior to death. Individually calculated Alb-M and log Alb-rCV values were averaged and plotted for the year before death. The vertical bars represent the 95% confidence intervals. The number of eligible subjects in each year is labeled in the figure.

**Fig 4:**
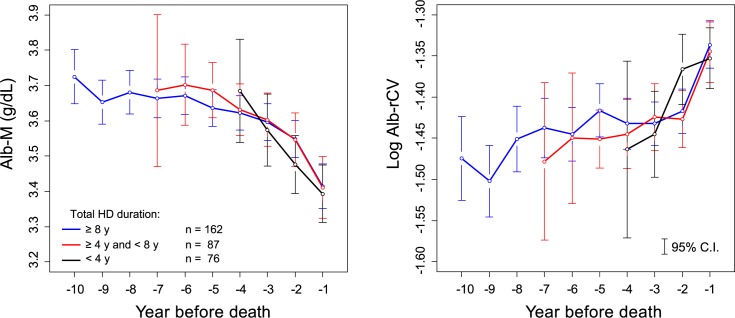
Duration of HD treatment and albumin dynamics prior to death. The before death subcohort was categorized into 3 groups according to the total duration of HD treatment, and the mean Alb-M or log Alb-rCV values of the groups were plotted for the year before death.

**Fig 5:**
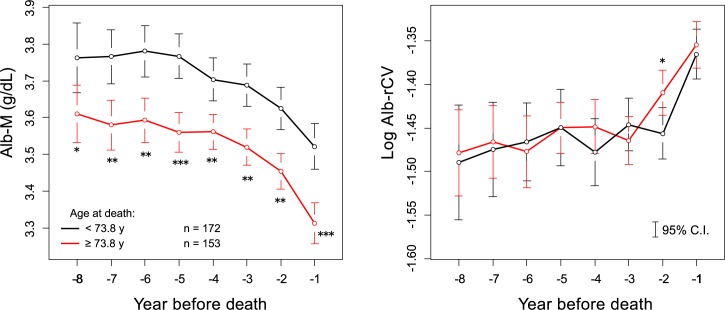
Age and albumin dynamics prior to death. The before death subcohort were divided into 2 groups according to the subject’s age at death (mean: 73.8 years), and the yearly mean values of both groups were compared using an unpaired t-test. *** *P* < 0.001, ** *P* <0.01, * *P* <0.05.

The levels of the annual mean albumin (Alb-M) and the log Alb-rCV in the years preceding death are shown in Fig 3. Alb-M demonstrated a visible downward trend, and this trend increased as death neared. On the other hand, albumin variability expressed as log Alb-rCV showed a contrasting upward trend. When the subjects were categorized according to their final HD duration (= survival time) into 3 groups (less than 4 years, between 4 to 8 years, and more than 8 years), the Alb-M or log Alb-rCV levels in the year before death were almost the same in all the groups (Fig 4). When the subjects were divided into 2 groups according to the age, both groups showed clearly different Alb-M levels in the years before death. However, the log Alb-rCV levels were comparable in both groups (Fig 5).

**Fig 6:**
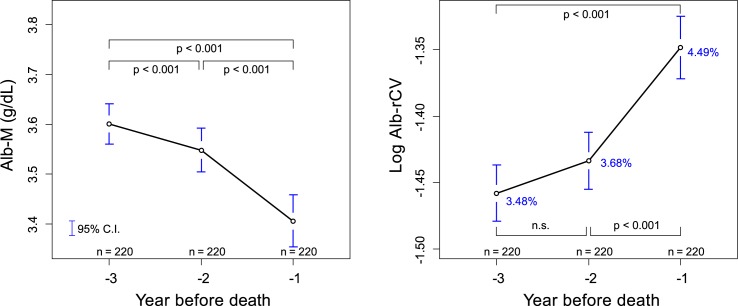
Annual comparison of Alb-M and Alb-rCV levels for the last 3 years before death. Among the before death subcohort, 220 patients who had survived and undergone continuous HD treatment at one of the study facilities for more than 3 years were selected. The mean Alb-M or log Alb-rCV values were then plotted for the year before death, and the statistical difference was examined using pairwise dependent t-test with Holm’s adjustment. The percentages written in blue are rCV values equivalent to a mean log Alb-rCV.

These yearly trends in Alb-M and log Alb-rCV, however, might be affected by changes in the patient population as a result of their transfer to another care facility or HD initiation. Therefore, 220 patients who continued to receive treatment at the study facilities for the entire 3 years before their death were selected, and their yearly Alb-M or log Alb-rCV levels were compared (Fig 6). The decrease in Alb-M was statistically significant between the third year before death (YBD) and the second YBD as well as between the second YBD and the first YBD. The increase in log Alb-rCV was significant between the second YBD and the first YBD.

### Serum albumin dynamics after HD initiation

**Fig 7:**
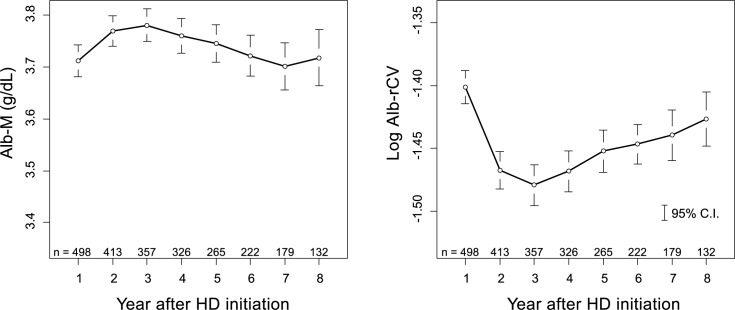
Yearly trends in albumin levels and variability after HD initiation. Individually calculated Alb-M and log Alb-rCV values were averaged for each year and plotted with their 95% confidence intervals. The number of patients included in each year is shown in the figures. The vertical scales of the graphs are matched with those in Fig 3 to simplify comparisons.

**Fig 8:**
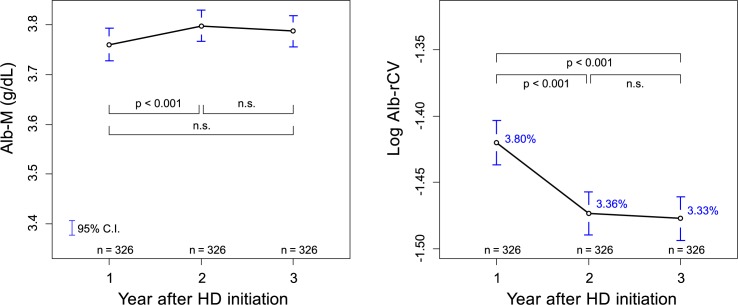
Annual comparison of Alb-M and Alb-rCV levels for the first 3 years after HD initiation. Among the post-HD initiation subcohort, 326 subjects who survived and continued to receive HD treatment at one of the study facilities for more than 3 years were selected. The mean Alb-M or log Alb-rCV values were then compared for the years after HD initiation using pairwise dependent t-test with Holm’s adjustment. The percentages written in blue are rCV values equivalent to a mean log Alb-rCV.

A similar analysis was performed for HD patients immediately after the initiation of HD. As shown in Fig 7, the average Alb-M initially increased and then decreased following the start of HD treatment. In contrast, the log Alb-rCV values showed the opposite movement. An analysis of 326 patients who survived and received HD treatment at one of the study facilities for the initial 3 years of their treatment showed a significant increase in Alb-M and a significant decrease in log Alb-rCV between the first and second year after HD initiation (Fig 8).

### Analysis with generalized additive mixed model

**Fig 9:**
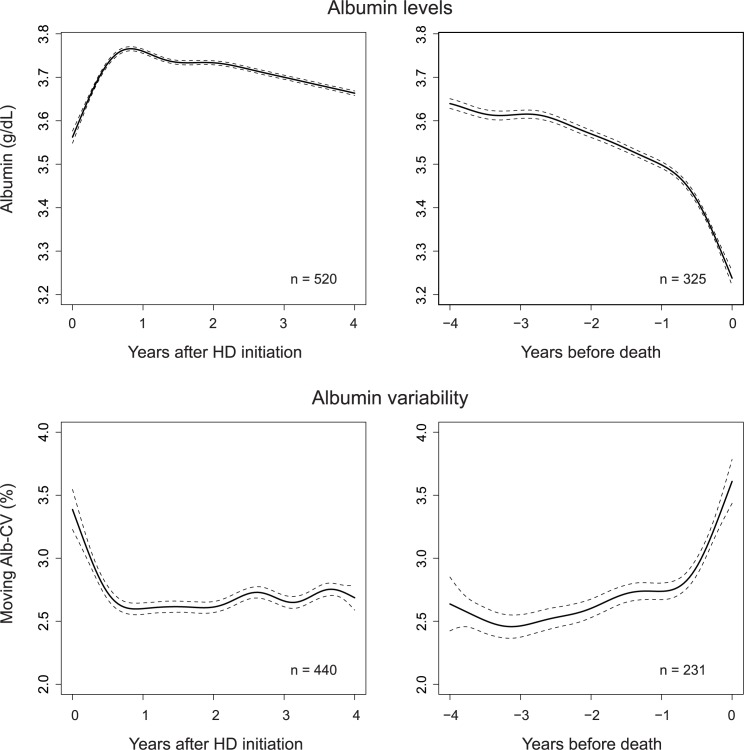
HD initiation and death-associated albumin dynamics estimated using generalized additive mixed models. Upper panels: All the albumin measurements for the post-HD initiation subcohort or the before death subcohort were fitted using a generalized mixed model with the time interval as a fixed variable and the subject ID as a random variable. The dotted lines represent the 95% confidence interval of the fixed term. Lower panels: The moving CV values were calculated using all the albumin measurements that had been recorded in January 2007 or thereafter; these values were fitted using a generalized mixed model in a similar manner. Note that the moving CV values were not log-transformed. The number of analyzed subjects is labeled in each panel.

While the analysis using yearly aggregated data provided solid corroboration for the occurrence of significant changes in the years before death and after HD initiation, it blunted shorter temporal changes. To identify changes on a shorter time scale, we supplemented the analysis with generalized additive mixed models (See Methods). These models clarified that both the albumin levels (Fig 9, upper panels) and the variability (Fig 9, lower panels) exhibited distinctive changes within 1 year after HD initiation and within 1–2 years before death.

### Changes during the maintenance period of HD treatment

Finally, we examined the albumin dynamics during the maintenance period of HD treatment, since the influences of HD initiation and of death were likely to be minimal at these time points. Based on the results provided above, we defined the maintenance period for each patient by excluding the first year of data following HD initiation and the 2 years of data proceeding the censored time from the entire study period, including some margins. Based on the Alb-M values calculated for every calendar year within the maintenance period, a linear regression model was applied to the remaining data for each patient.

**Fig 10:**
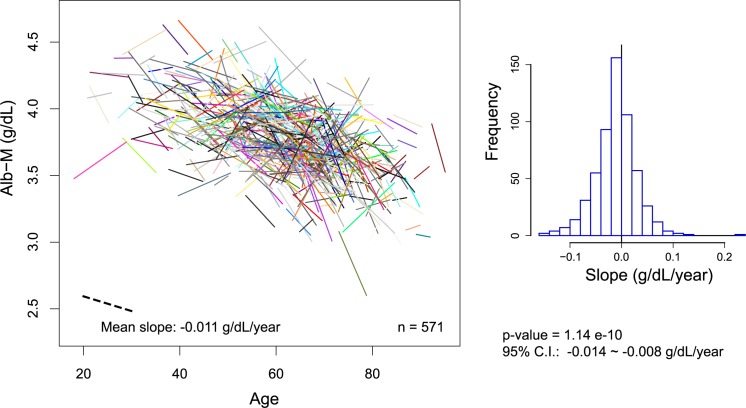
Overall Alb-M movement during the maintenance period of HD treatment. For each subject, a regression line was calculated from a set of yearly Alb-M values, and the line was then positioned according to patient age (left). The distribution of the slopes is shown in the histogram (right).

To identify intra-individual longitudinal changes in Alb-M and the inter-individual association with age simultaneously, the regression lines were aligned according to each subject’s age (Fig 10, left panel). As shown in the right panel, the mean regression slope for Alb-M was relatively small (-0.011 **±** 0.04 g/dL/year) but was significantly less than zero. If we regard the Alb-M value at the midpoint of the regression line as the overall albumin level for each patient, an association with patient age was apparent.

**Fig 11:**
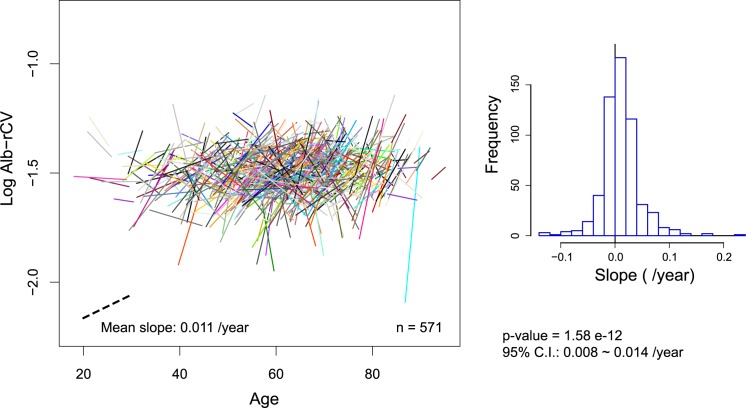
Overall Alb-rCV movement during the maintenance period of HD treatment. The regression lines for the log Alb-rCV values are displayed in the same manner as that used for Alb-M in Fig 10.

The change in log Alb-rCV was analyzed in a similar fashion (Fig 11). The mean slope of the regression lines for log Alb-rCV was significantly higher than zero (0.011 **±** 0.04 /year) and was equivalent to an increase in the rCV value from 3.1% to 3.7% over 10 years. The Alb-rCV tended to increase with time within individuals, though their midpoint Alb-rCV values (overall variability levels) were not correlated with patient age.

### Mutual associations among parameters of albumin dynamics and demographics

The correlations among the overall levels of Alb-M and log Alb-rCV, their slopes, and demographic parameters during the maintenance period were examined (Table 1). Alb-M was negatively correlated with log Alb-rCV and its slope (upward trend). Subjects with a decreasing trend for Alb-M tended to have an increasing trend for Alb-rCV. Concerning demographic factors, the Alb-M level was associated with a younger age, a male sex, and a non-diabetic status, whereas the log Alb-rCV level was poorly associated with these factors. The slope of log Alb-rCV was nevertheless weakly associated with age. The length of the maintenance period was associated with a higher Alb-M level, a higher Alb-M slope (i.e., a slower Alb-M decline), and a lower Alb-rCV level.

**Table 1:** Correlations among parameters of albumin dynamics and demographic factors during the maintenance period.

		Alb-M		Log Alb-rCV	
		Level^a^	Slope^b^	Level^a^	Slope^b^
**Alb-M**	**Level**^a^	-	0.057	-0.217***	-0.115**
	**Slope**^b^		-	-0.079	-0.188***
**Log Alb-rCV**	**Level**^a^			-	0.014
	**Slope**^b^				-
**Age**^a^		-0.571***	-0.037	0.049	0.097*
**Gender**^c^		-0.187***	0.103*	0.038	0.046
**Diabetic status**^c^		-0.102*	0.002	0.062	-0.041
**HD duration**^a^		-0.025	0.071	-0.121**	-0.073
**Maintenance period**^d^		0.202***	0.112**	-0.204***	-0.060

The numbers represent the Pearson’s correlation coefficients; *P*-values < 0.05 were printed in boldface.

^a^ Value corresponding to the middle point of the regression line for each subject

^b^ Slope of the regression line for each subject

^c^ Categorical variables (Gender and diabetic status) were treated as continuous variables (male = 0, female = 1, non-diabetic = 0, and diabetic = 1) to identify positive or negative relationships between the variables. In combinations with other continuous variables, the *P*-value was obtained using the Welch *t*-test.

^d^ Length of the maintenance period for each subject

*** *P* < 0.001

** *P* < 0.01

* *P* < 0.05

## Discussion

### Albumin levels

Serum albumin is known to be a strong predictor of outcomes in various conditions and has been proposed as a marker of illness [23–27]. So far, several studies have examined longitudinal changes in its levels in chronic HD patients. A small and transient increase in albumin levels following the initiation of HD treatment [28–30] and a more distinctive and accelerating decrease before death [29,31,32] have been reported. Although these results could be affected by the existence of oxidized albumin [33], such peculiar changes in albumin levels during the initial and terminal phases of HD treatment are consistent with our observations and seem to be universal phenomena. On the other hand, albumin dynamics between these two phases are not yet well understood. Rocco et al. reported a mean albumin decline of 0.10 g/dL over 3 years among HD patients who survived for more than 3 years [34], and den Hoedt et al. showed an annual albumin decline of 0.08 g/dL during a 6-year follow-up period [35]. These values, however, might be influenced by changes associated with the initiation of HD and with death. By using data from subjects who had received HD treatment for relatively long periods of time, we were able to isolated a maintenance period (interquartile range: 5.2–11.0 years) within the entire study period. The mean rate of albumin decline during this period was small (-0.011 g/dL/year) but was significantly lower than zero. This finding indicates that serum albumin levels tend to decrease with time even among patients who have been stably receiving HD treatment for long periods of time.

### Albumin variability

The intra-individual variability of various laboratory parameters has often been estimated using the CV and has also been called “biological variation” (BV). In a review article on BV, the subjects' age and sex and the sampling interval were reported to have little influence on BV estimates [36]. The BV levels in patients with chronic diseases have been compared with those of healthy people in several studies, with various results [8–11,37]. Among them, Holzel reported high BV levels for several parameters in patients with chronic renal failure, chronic liver disease, or insulin-dependent DM, compared with healthy individuals. In addition, Fraser et al. compared the BV levels in healthy elderly people with those in young people for various parameters and showed divergent results depending on the selected parameter [38]. These results, however, seem to be inconclusive, since most studies on BV were cross-sectional and based on a limited number of samples from relatively small cohorts.

In this study, we presented the longitudinal changes in albumin variability for the first time. Alb-rCV temporarily decreased following the start of HD treatment. However, the movement was soon reversed, and the upward trend began to accelerate at one year or more before death. The Alb-rCV apparently moved in a manner opposite to that of the albumin levels throughout the course of HD treatment. During the maintenance period, the slopes as well as the levels of log Alb-rCV were negatively associated with the Alb-M levels. These results suggest that both a high albumin variability and a low albumin level develop in parallel with deteriorating health conditions.

Although the movements of Alb-M and Alb-rCV were closely related, as stated above, differences became evident if the relationships between the overall levels of these parameters and demographic factors during the maintenance period were examined (See Table 1, and Figs 10 and 11). While Alb-M was strongly associated with age and moderately associated with gender and diabetic status, log Alb-rCV was poorly associated with these factors. These relationships are consistent with our previous cross-sectional analysis [7]. A similar situation for age was also observed in the years prior to death (Fig 5). Thus, although albumin variability was poorly correlated with age in inter-individual comparisons, it still tended to increase with age on an individual basis. In other words, albumin variability is associated with individual “aging” rather than chronological age. Similar to albumin variability, frailty is not determined simply by chronological age, but mostly progresses with aging in individuals.

### Homeostasis and albumin dynamics

Homeostasis is thought to be maintained by multiple overlapping regulatory networks in the body, and many investigators have recognized the dysregulation of homeostasis as being a fundamental component of aging and frailty [15,16,39–43]. Fried and her collaborators have tested this concept in several papers. For example, Kalyani et al. performed oral glucose tolerance tests in elderly women and reported an abnormal (diabetic) response in a frail group [44]. Cohen et al. estimated an individual’s deviation from a normal status using the Mahalanobis distance for multiple laboratory parameters and showed associations with mortality, frailty and chronic diseases [45]. Furthermore, Cohen and others demonstrated that physiological dysregulation as assessed using the Mahalanobis distance increases with age [46,47].

Given that engineering control systems can mimic physiological regulatory mechanisms [48], dysregulation can emerge in at least 3 forms: i) an aberrant value, ii) an inadequate response to disturbance, and iii) a wider variability. From this point of view, these studies addressed the first two forms. We believe that the third form, variability, could be used as a measure of physiological dysregulation in a population-level analysis [49]. This idea is consistent with the following findings: (a) albumin variability increases with age on an individual basis, (b) this trend accelerates before death, (c) variability of several laboratory parameters predicts mortality, and (d) a high variability is often associated with several frailty-related adverse factors [7]. In our preceding study, we observed that the variabilities of different laboratory parameters were correlated with each other when almost all the combinations of parameters were compared [7]. The hypothesis mentioned above could provide a possible interpretation for this enigmatic finding as well. To maintain constant values for each parameter, multiple regulatory mechanisms must work together inside the body, and it is highly likely that some of these regulatory mechanisms are shared by different parameters. A functional loss affecting shared mechanisms might cause higher variability in all the involved parameters, and this feature might manifest as correlations among the variabilities of these parameters.

Greater variability can be caused by not only a loss of homeostatic capacity, but also enhanced disturbances (i.e., internal and external stressors). Potential stressors presumably include physical activities, eating, climate, acute illness and medication, and evaluating the magnitudes of these stressors is obviously difficult. From a logical perspective, the CV (or rCV) should be used as a measure of homeostatic dysregulation only in population-based analyses with sufficient numbers of subjects. In addition, since this study was based solely on data from chronic hemodialysis patients, whether the results are applicable to the general population remains unknown. Despite these limitations, we think that the CV or other estimators of variability could be valuable tools for probing disordered homeostatic control in various pathological conditions.

In the present study, we focused on time-dependent changes in albumin dynamics. For the maintenance of life, however, the homeostatic regulation of broader body constituents is necessary. Therefore, to understand the significance of variability in a precise manner, the present analysis should be expanded to include other parameters.

## Conclusions

Except for the period after the initiation of HD, serum albumin variability exhibited an upward trend in chronic HD patients, and this trend accelerated as death approached. These aging and death-associated changes in albumin variability seemed to parallel the presumed decline in homeostatic capacity and healthy conditions. Further analysis of the variability of albumin and other parameters may contribute to the advancement of knowledge regarding the pathophysiology of aging and diseases.

## Supporting information

S1 Fig Final hemodialysis duration in the total cohort. (EPS)
